# Cobalt chloride-stimulated hypoxia promotes the proliferation of cholesteatoma keratinocytes via the PI3K/Akt signaling pathway

**DOI:** 10.7150/ijms.60617

**Published:** 2021-08-02

**Authors:** Chen Zhang, Min Chen, Qi Tao, Zhangcai Chi

**Affiliations:** 1 Department of Otolaryngology, Eye & ENT Hospital, Fudan University, 83 Fenyang Road, Shanghai, China.; 2 NHC Key Laboratory of Hearing Medicine (Fudan University), Shanghai, 200031, PR China.; 3 Nursing Department, Eye & ENT Hospital, Fudan University, 83 Fenyang Road, Shanghai, China.

**Keywords:** hypoxia, cholesteatoma, HIF-1α, cell proliferation, PI3K/Akt signaling pathway

## Abstract

Herein, we purposed to explore whether hypoxia triggers proliferation of cholesteatoma keratinocytes via the PI3K-Akt signaling cascade. Cells were inoculated with different concentration of CoCl_2_. The proliferation and cellular HIF-1α, p-PDK1 and p‑Akt expression levels of cholesteatoma keratinocytes were assessed *in vitro*. Hypoxia escalated cell proliferation via upregulating p-PDK1 and p‑Akt expressions. Specific inhibitor of the PI3K-Akt signaling cascade, LY294002 markedly inhibited the expression of p‑Akt and significantly reduces the hypoxia‑induced proliferation of cholesteatoma keratinocytes. Our data provides research evidence confirming that hypoxia participates in the onset and progress of cholesteatoma.

## Introduction

Middle ear cholesteatoma, a disease that manifests by both the overgrowth of hyper-keratinized squamous epithelium along with its aggressive clinical course with extensive bone destruction potentially resulting in severe complications, such as refractory otorrhea, balance dysfunction, vertigo, and progressive hearing loss 1. This disease always demands surgical therapy; however, it is reported that the recidivism rate of this disease was in a wide range, from 4 to 61% 2. Therefore, the long-term eradication of cholesteatoma is still presenting a challenge for experienced otologists. The scientific pathogenesis of cholesteatoma has been investigated, with progress in studies of many factors, as well as receptors of the cholesteatoma keratinocyte^3^; however, the molecular mechanism responsible for the pathogenesis of cholesteatoma remains unknown.

The theory of a combination of invagination and proliferation which described by Sudhoff and Tos 4, seems to be applicable to elucidate the retraction pocket in cholesteatoma formation. Based on this observation, the tympanic membrane retraction pocket is commonly secondary to the chronic dysfunction of Eustachian tube. The interruption of aeration through the Eustachian tube exposes the middle ear cavity to reducing pressure and reducing oxygenation; causing hypoxia along with hypercapnia of the middle ear mucosa 5.

It is demonstrated that the hypoxic status in the tympanic cavity can lead to eardrum retraction pockets, followed by cholesteatoma formation. Thus, for long hypoxia has believed as one of the primary factors promoting the onset and progress of cholesteatoma 6, 7. In the hypoxia status, (HIF-1α) hypoxia-inducible factor 1α is activated and subsequently controls the expression of various genes necessary for mounting cellular responses to hypoxia 8. Besides, the HIF-1α transcription factor is distributed ubiquitously, and has been documented to participate in numerous processes constituting cell proliferation, metabolism, angiogenesis, cell transformation and cell senescence 9. Increased HIF-1α contents have been documented in cholesteatoma tissues, and recurrent cholesteatomas result in an elevated degree of hypoxia than ears without surgery patients, suggesting that the cholesteatoma has been recognized as a hypoxic tissue and HIF-1α plays a vital role in the proliferation ability of cholesteatoma epithelium 6. However, studies on HIF-1α expression in cholesteatoma and its relationship with the pathological changes such as cholesteatoma keratinocyte proliferation on one hand, and with the intrinsic signaling pathways on the other hand, have not been fully understood.

The serine/threonine kinase Akt also referred to as protein kinase B has a vital role in cell growth, differentiation, as well as survival 10. The phosphoinositide 3-kinase (PI3K)-dependent signaling cascade activates Akt in response to diverse stimuli consisting of certain cytokines and growth factors 11. In the hypoxia status, the PI3K-Akt signaling cascade is activated in distinct cells, such pulmonary arterial smooth muscle, porcine coronary artery endothelial, A549 cells, as well as rat hepatocytes, while other cells; consisting of HEK293T, PC‑3, U373, COS‑7, and 3T3 cells do not exhibit elevated Akt phosphorylation levels 12, 13. Previous investigations have demonstrated that the PI3K-Akt-Cyclin D1 signaling cascade is activated in cholesteatoma epithelium and has a critical role in cholesteatoma keratinocytes hyperproliferation 1. Previous studies revealed that hypoxia increased cell proliferation for cholesteatoma keratinocytes 2. However, the relationship between the PI3K-Akt signaling cascade and hypoxia‑induced proliferation in cholesteatoma keratinocytes remains unclear.

Herein, we explored whether PI3K-Akt signal cascades are activated by hypoxia and, if so, to elucidate their function and signaling cascades in cholesteatoma keratinocytes. Our data illustrated that hypoxia triggered transient activation of the PI3K-Akt survival signaling cascades via a PKC-dependent mechanism. These signals were linked to proliferation of cholesteatoma keratinocytes when exposed to hypoxia.

## Materials and methods

### Materials

LY294002 was supplied by MedChem Express (NJ, United States). All the reagents used herein were of analytical grade.

### Tissue Sample and immunofluorescence

Tissue samples were acquired from 10 study participants (five participants with primary acquired cholesteatoma and five participants with normal external canal skins) for immunofluorescence and Western blot. The Research Ethics Committee of the Eye and ENT Hospital of Fudan University approved the study. Moreover, the study subjects gave written informed consent. After surgery, these cholesteatoma samples were split into two parts for immunohistochemical analyses and cell culture. The immunohistochemical samples were fixed with 4% PFA for 24 hours and then paraffin-embedded. Afterwards, 5-mm slices were employed for immunofluorescence as documented previously. Graded alcohol was employed to deparaffinize and rehydrate the sections, followed by blocking for one hour in 10% normal goat serum. Next, the samples were rinsed, and then inoculated with primary antibodies against HIF-1α antibody (Cat No GTX628480, Genetex, Cambridge, MA, USA; 1:200), HIF-1α antibody (Cat No GTX127309, Genetex, Cambridge, MA, USA; 1:200) and Pan-keratin (Cat No 4545, CST, Danvers, MA, USA; 1:200). Thereafter, the samples were inoculated with Alexa 546-conjugated or Alexa 488‑conjugated goat anti‑rabbit/mouse antibodies for one hour at RT (room temperature). Then, staining of the sections with DAPI was performed. Lastly, Axioskop microscope (Carl Zeiss, Oberkochen, Germany) was employed to image the samples.

### Cell culture and stimuli

A previously documented protocol was employed to isolate and characterize the cholesteatoma keratinocytes 14. Concisely, cholesteatoma tissues were acquired from study subjects via surgical resection and hand carried to our laboratory. A scissors was used to slice the tissues into small slices. Afterwards, the tissue slices were inoculated with 200 U/ml collagenase IV (Cat No C5138, Sigma Aldrich, St. Louis, MO, USA) overnight at 4℃ to dissociate the tissues. Next, HBSS was employed to rinse the tissues twice, followed by spinning at 1,500 rpm for five minutes. The supernatant was discarded and the pellet inoculated with KSFM medium (Cat No 17005042, Invitrogen, Carlsbad, CA, USA) enriched with 500 u/ml streptomycin/penicillin (Cat No 15070063, Invitrogen, Carlsbad, CA, USA), and grown under 5% CO2 and 37℃ conditions. After every three days, we changed the KSFM media along with the antibiotics. The cell cultures of between the 3^rd^ and 4^th^ passages were used in the assays. In all the assays, unless indicated, 1x10^5^ cells/cm^2^ were planted in culture plates and allowed to grow to a confluence of between 70% and 80% for use. Pretreatment with inhibitor, LY294002 as a PI3K inhibitor (Cat No HY-10108, MedChem Express, NJ, United States)), was accomplished via the introduction of the inhibitor one hour before induction in the presence or absence of hypoxia. Hypoxic conditions were introduced by treating cells with 50 uM to 200 uM concentrations of CoCl_2_ to in a cell incubation chamber (Don Whitley Scientific Ltd., Shipley, UK) for the specified time.

### EdU staining proliferation assay

The EdU imaging kit (Cat No C10638, Invitrogen, Carlsbad, CA) was employed to assess cell growth after the specified treatments as described by the manufacturer. In brief, 5*10^3^ Cholesteatoma keratinocytes/well were inoculated in KSFM medium in the Lab-Tech chamber slide (Nalge Nunc International, Cambridge, MA, USA) until they attained a confluence of 70-80%. These cells were subsequently inoculated with LY294002 (10 μM) one hour before exposure to indicated oxygen conditions for specified period. After that, the cells were fixed with 4% PFA for 15 minutes, followed by permeabilization for another 15 minutes using 0.3% Triton X-100 dissolved in PBS. Afterwards, we inoculated the cells in a Click mixture (Click reaction buffer, reaction buffer additive, CuSO4, and Alexa Fluor® 555 Azide) for 30 minutes in the dark. The cells were then inoculated with 5 μg/mL Hoechst 33342 for 10 minutes for DNA staining. Lastly, a fluorescent microscope was employed to image the cells, and the proportion of EdU-positive cells determined.

### Western blot analysis

After, the specified treatments, ice-chilled PBS was employed to wash the PAFs twice. Afterwards, 0.15 ml cell lysis buffer (Cat No P0013, Beyotime Institute of Biotechnology, China) enriched with protease inhibitor was employed to lyse the cells for 15 minutes. Next, the BCA kit (Cat No, Beyotime Institute of Biotechnology, China) was employed to quantify the proteins. Subsequently, 30 μg/lane proteins were fractionated on a 10% SDS-PAGE gels. The fractionated proteins were blotted onto PVDF membranes (300 mA for one hour). The membranes were inoculated with the primary antibodies and incubated at 4℃ overnight. Thereafter, the membranes were inoculated with the secondary peroxidase-labelled antibody for one hour. The protein bands were detected via enhanced chemiluminescence and exposure to ECL Hyperfilm (GE Healthcare). NIH Image 1.63 software was employed to quantify the densitometry of bands. Beta-actin served as the normalization standard. The primary antibodies used included anti-phospho-PDK1 (1:1,000), anti-protein kinase B (AKT) (1:1,000) and anti-phospho-AKT (Ser 473; 1:1,000) (All from Cell Signaling Technology, Danvers, MA, USA). Antibodies against β-actin (1:1,000) and HIF-1 α (1:2,500) were from Genetex, Inc. (Genetex, CA, USA).

### Statistical analyses

The data are given as the mean ± standard deviation (SD). Paired Student's t-test was implemented to compare two groups. P < 0.05 signified statistical significance.

## Results

### Immunolocalization of HIF-1α

**Figure 1:**
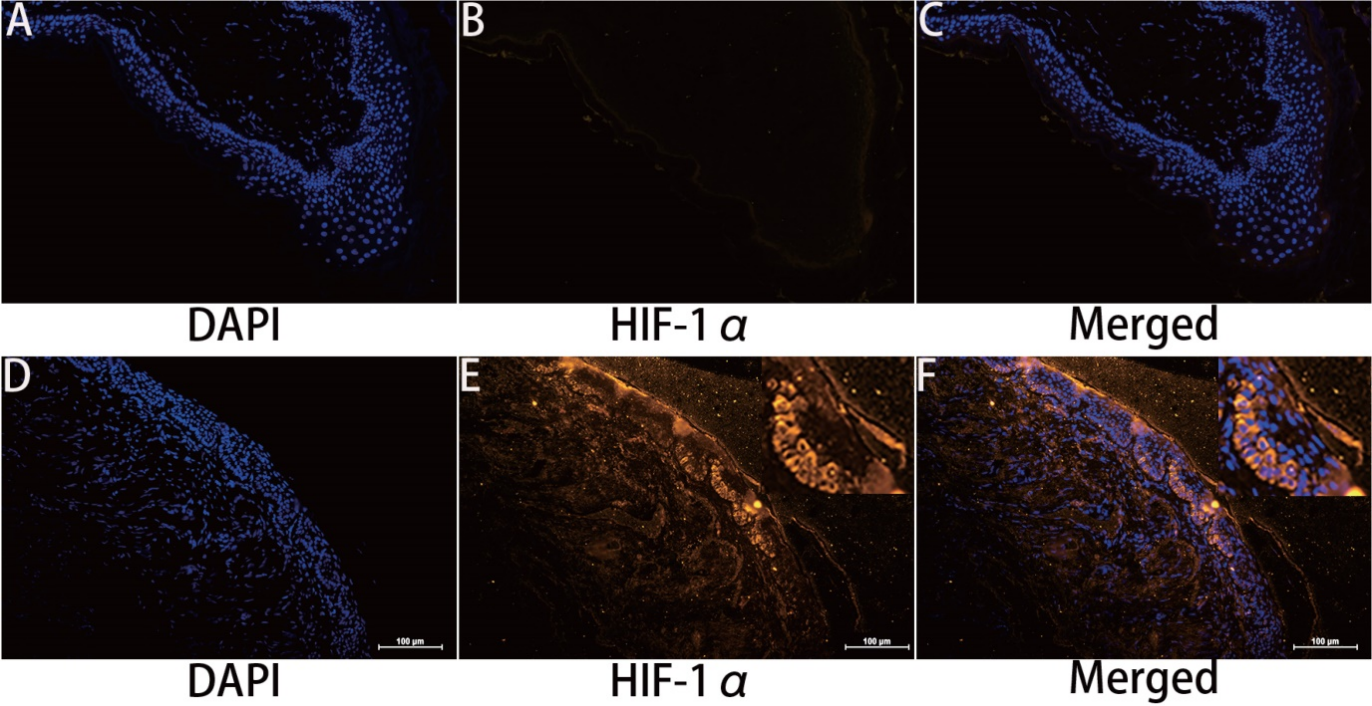
Immunohistochemical staining for hypoxia inducible factor 1α. HIF-1 α is scantily expressed in external auditory canal skin (A-C). HIF-1 α is expressed in the cytoplasm of cholesteatoma keratinocytes in the parabasal, as well as basal layers of cholesteatoma epithelium (D-F). The highest staining intensity is evident in basal layer keratinocytes (magnification, ×200).

**Figure 2:**
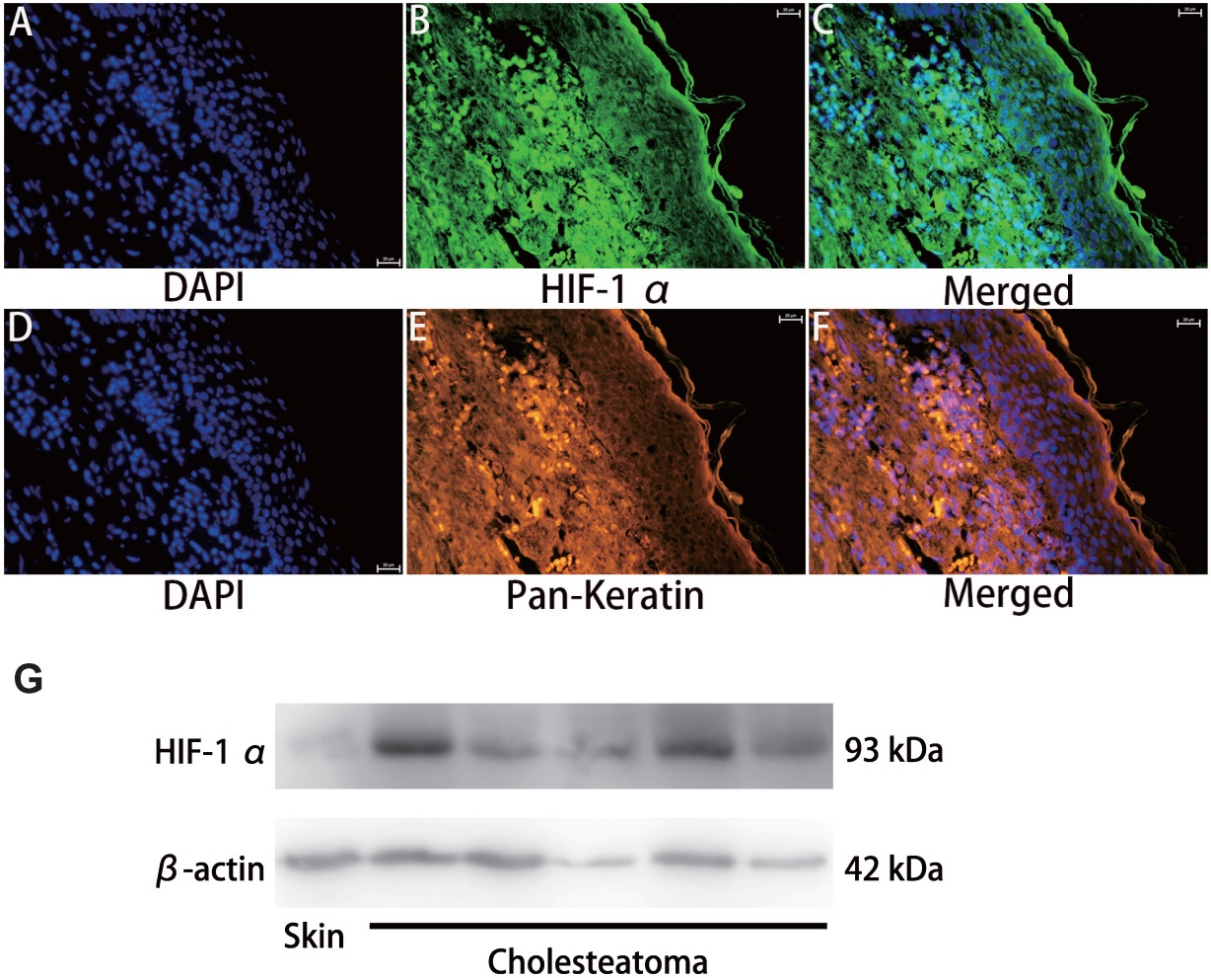
Double immunofluorescence staining and western blotting analyses of hypoxia inducible factor 1α and Pan-keratin in human cholesteatoma tissues. HIF-1α (A-C) and Pan-keratin (D-F) was present in the cytoplasm of cholesteatoma keratinocytes in the cholesteatoma epithelium (magnification, ×400). (G) Western blotting images of HIF-1α.

HIF-1α expression in cholesteatoma tissue was remarkably upregulated relative to the normal skin according to the results of the immunofluorescence experiments (Fig. 1). In the healthy skin, scanty staining of HIF-1α was seen (Fig. 1A-C). In cholesteatoma tissue, HIF-1α was obviously expressed in the cytoplasm of the basal and parabasal cell layers (Fig. 1D-F). Next, we performed a co-stained of intrinsic hypoxia marker HIF-1α (Fig. 2A-C) and a keratinocyte marker Pan-keratin (Fig. 2D-F) in the cholesteatoma samples, and demonstrated that the staining of HIF-1α and Pan-keratin was present in cytoplasm. In addition, we observed a co-expression of Pan-keratin and HIF-1α in the epithelium of cholesteatoma, strongly suggesting that HIF-1α is upregulated in cholesteatoma keratinocytes.

To further confirm the effective expression of HIF-1α, we performed western blotting analysis. Results showed that the protein expression of HIF-1α in cholesteatoma tissues was significantly higher than that in the normal skin (P<0.05) (Fig. 2G).

### CoCl_2_-simulated hypoxia upregulates HIF‑1α expression in cholesteatoma keratinocytes

**Figure 3:**
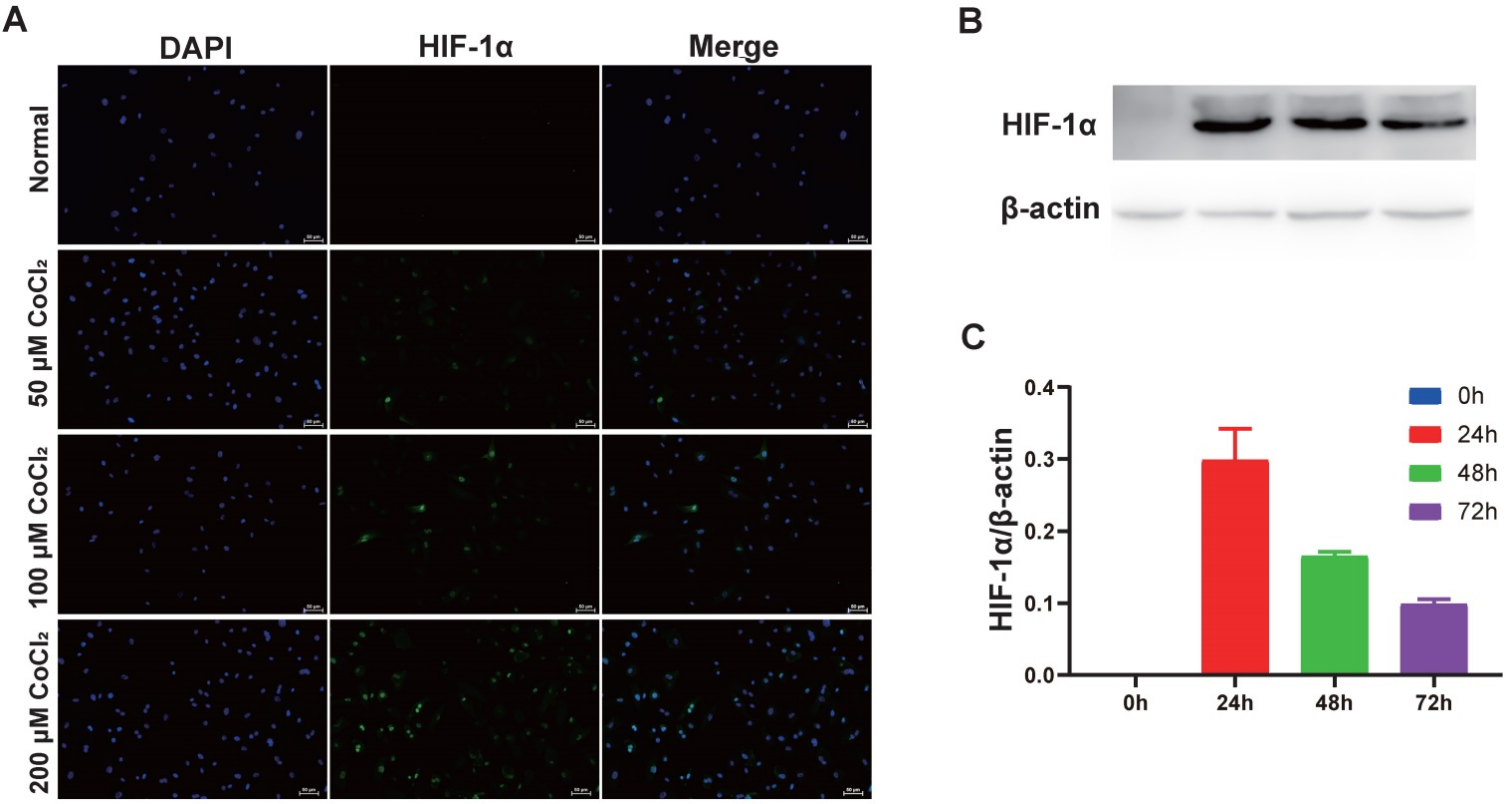
Hypoxia increases HIF‑1α expression in cholesteatoma keratinocytes. (A) Cells were inoculated with 50 μM、100 μM and 200 μM CoCl_2_ for 24 h and the HIF‑1α protein was stained and observed by fluorescent microscopy (magnification, x200). (B) Cholesteatoma keratinocytes were inoculated with 50 μM CoCl_2_ for 0, 24, 48, 72 h and HIF‑1α expression was assayed by western blotting. (C) Results represent three independent experiments. β-actin served as the loading control. Data are given as mean ± standard error of the mean.

CoCl_2_ triggers HIF-1α protein aggregation in numerous kinds of cells 15. To explore if CoCl_2_ influenced HIF-1α expression, cholesteatoma keratinocytes were inoculated with CoCl_2_ for different times and HIF‑1α protein expression was assessed via western blotting. As illustrated in Fig. 3A, the increase in HIF‑1α protein expression in CoCl_2_-treated cells was observed by fluorescent microscopy. Additionally, the HIF‑1α protein expression increased dose-dependently after CoCl_2_ treatment. Based on these data, the stimulation of HIF‑1α under 200 μM CoCl_2_ was optimal. To explore if HIF‑1α expression occurred in a time‑dependent approach, the cells were inoculated with 50 μM CoCl_2_ for 0, 24, 48 and 72 hours, and HIF‑1α expression was assessed via western blotting. As indicated in Fig. 3B-C, HIF‑1α expression was higher under hypoxia conditions (50 μM CoCl_2_), peaked at 24 hours and reduced after 24 hours, implying that internal mechanism repressed further increase in HIF‑1α expression. Thus, hypoxia induced by CoCl_2_ treatment was critical in upregulating HIF‑1α protein expression in cholesteatoma keratinocytes.

### CoCl_2_-simulated hypoxia enhances the proliferation of cholesteatoma keratinocytes

**Figure 4:**
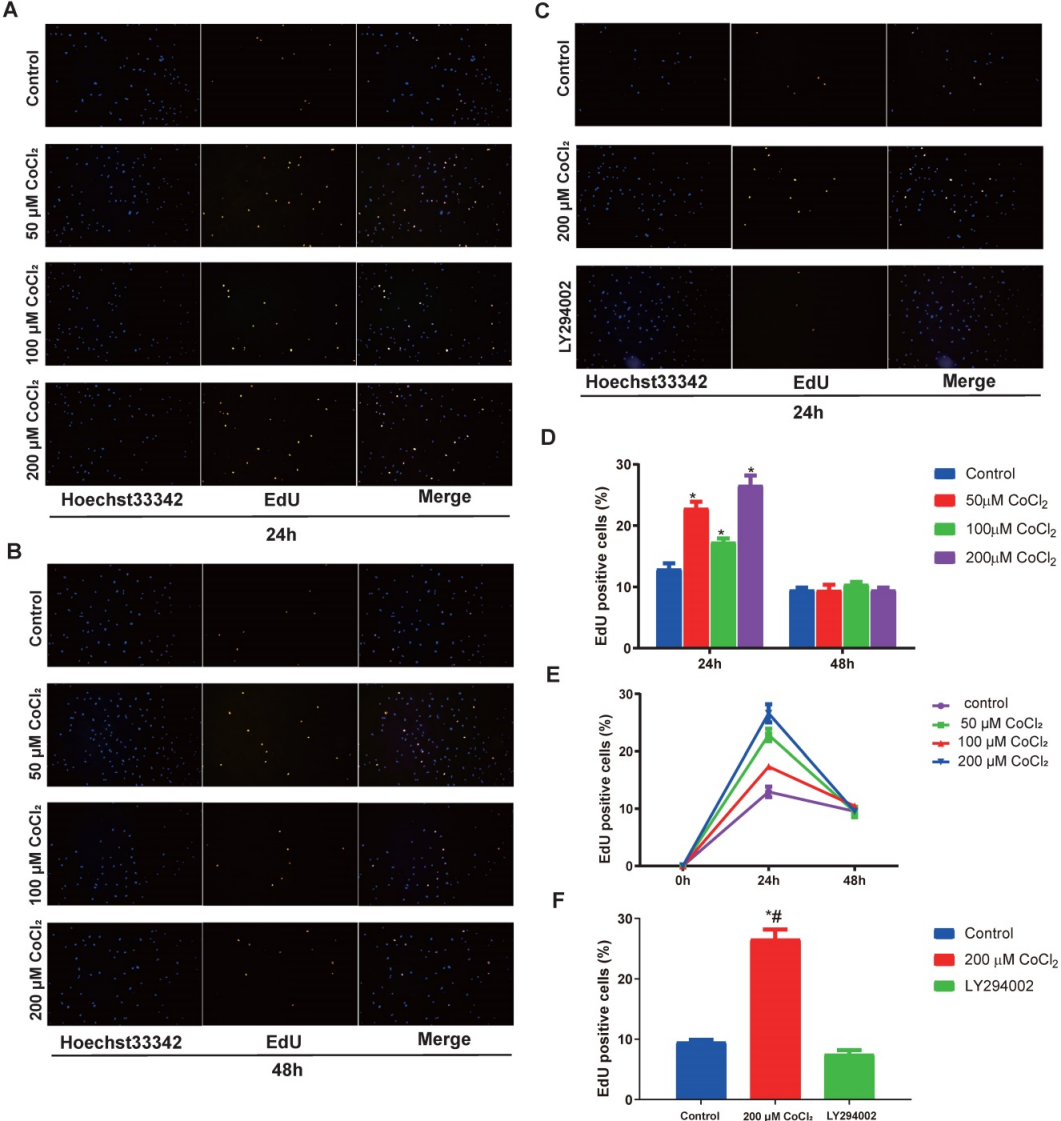
Hypoxia‑induces the proliferation of cholesteatoma keratinocytes *in vitro*. (A-C) The proliferation of cholesteatoma keratinocytes was explored using EdU assay after treatment as specified for 24 and 48 h (n=5). (D) Hypoxia enhanced cell proliferation in a concentration‑dependent approach after CoCl_2_ treatment for 24 h. The optimal concentration of CoCl_2_ is 200 μM (n=5). (E) Hypoxia enhanced cell proliferation in a time‑dependent approach after CoCl_2_ treatment, peaked at 24 h and decreased after 24 h (n=5). (F) The proliferation of cholesteatoma keratinocytes was inhibited followed treatment with LY2940002. *P<0.05.

To explore the function of hypoxia on cell proliferation, the cholesteatoma keratinocytes were inoculated with varied concentrations of CoCl_2_ for 1‑48 h and the proliferated cell number was quantified using an EdU assay. As indicated in Fig. 4A and 4D, hypoxia enhanced cell proliferation in a concentration‑dependent approach after CoCl_2_ treatment for 24 h. In addition, the promotion of proliferation under severe levels of hypoxia (200 μM CoCl_2_) was more marked. The number of EdU-positive cell increased in hypoxia, peaked at 24 h and decreased after 24 h, suggesting prolonged hypoxia inhibited cell proliferation (Fig. 4E). In conclusion, hypoxia, to some extent, enhances the proliferation of cholesteatoma keratinocytes.

### CoCl_2_-simulated hypoxia promotes the proliferation of cholesteatoma keratinocytes via the phosphoinositide 3‑kinase (PI3K)/Akt pathways

**Figure 5:**
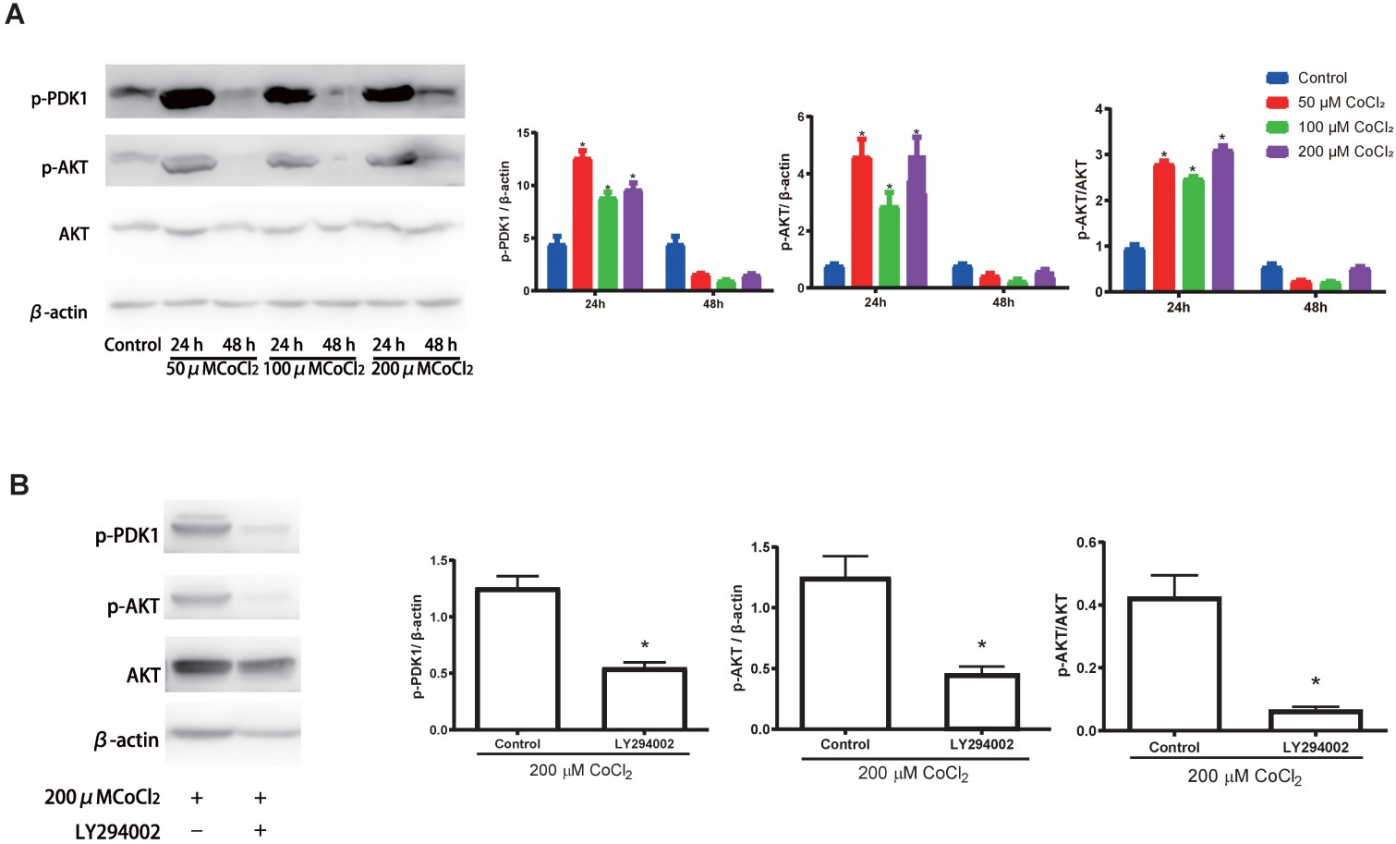
Hypoxia activates the PI3K-Akt signaling cascade in cholesteatoma keratinocytes. (A) Western blotting assay of specified proteins in cholesteatoma keratinocytes after inoculation with 50 μM、100 μM and 200 μM CoCl_2_ for 24 h and 48 h. (B) Western blotting assay of the specified proteins in cholesteatoma keratinocytes after inoculation with LY294002 and hypoxia as specified for 24 h. *P<0.05.

Previous investigations have documented that the PI3K-Akt cascades play an important role in modulating the proliferation of cholesteatoma. Herein, we explored whether this signaling cascade was involved in modulating the proliferation of cholesteatoma keratinocytes under hypoxic status. The cholesteatoma keratinocytes were inoculated with varied CoCl_2_ concentrations and the contents of PDK1、Akt and p‑Akt were assessed with western blotting. Based on the western blot analysis results (Fig. 5A), hypoxia remarkably elevated the activation of PDK1and p‑Akt. To explore if the hypoxia‑triggered activation of Akt particularly modulated the cell proliferation of cholesteatoma keratinocytes, the PI3K-Akt cascade was repressed by LY294002, a suppressor of PI3K. The LY294002 (10 μM) remarkably reduced the activation of p‑Akt (Fig. 5B) and inhibited the proliferation of cholesteatoma keratinocytes (Fig. 4C, 4F). These data illustrated that hypoxia‑triggered proliferation of cholesteatoma keratinocytes is dependent on the PI3K-Akt pathways.

## Discussion

In this study, we have provided evidence that HIF‑1α, act as hypoxic-associated molecule is highly expressed in human cholesteatoma tissues. In line with previous works 6, our result confirm that cholesteatoma is a hypoxic tissue. Evidently increased staining was reported in the keratinocytes located in the basal layer of the cholesteatoma epithelium. This result suggests that hypoxia participates in the control of proliferation of cholesteatoma epithelial cells and act as a central mediator in the formation of the hyperplastic epithelium.

As mentioned above, cholesteatoma is a hypoxic tissue; however, the impact of hypoxia on cholesteatoma keratinocytes has not been thoroughly investigated. In this study, we inquired the effects of CoCl_2_-simulated hypoxia on the cholesteatoma keratinocytes. Because CoCl_2_ strongly stabilizes HIF-1/2α under normoxia, CoCl_2_-induced chemical hypoxia becomes one of most commonly used models to simulate hypoxia 16. Hypoxia can variably affect cell growth via diverse mechanisms in different cell types. Hypoxia inducible factors (HIFs) are understood to be the master regulators of the cellular response to hypoxia 8. The HIF-1/2 are dimer consisting of HIF-1/2a protein subunit and the beta subunit aryl hydrocarbon receptor nuclear translocator (ARNT) or HIF-β protein subunit 8. During normoxia, PHD enzymes can hydroxylate HIF-1/2a with help of oxygen, iron (Fe^2+^) and a-ketoglutarate, followed by polyubiquitination by the VHL complex and subsequent proteasomal degradation 8. It is well known that the mechanism by which CoCl_2_ stabilizes HIF-1/2a is the displacement of Fe^2+^ by Co^2+^ in PHDs active site provoking inhibition of these key enzymes 16. Thus, the CoCl_2_-induced hypoxia model is reliable for us to analyze the effect of hypoxia on cholesteatoma keratinocytes.

Herein, we found that culture of cholesteatoma keratinocytes isolated from cholesteatoma tissues under hypoxic conditions caused a concentration-dependent promotion of keratinocyte proliferation and increased HIF-1α expression under hypoxia conditions. In addition, we found that cholesteatoma keratinocytes exposed to hypoxia for 24 hours were still able to grow, however at a slower rate relative to cells grown in normoxia. This lower cell count reported under conditions of prolonged hypoxia does not appear to be due to apoptosis, because cell showed no apoptotic morphological changes. Thus, hypoxia can enhance proliferation and prolonged hypoxia can induce a state of quiescence in cholesteatoma keratinocytes.

CoCl_2_-simulated hypoxia actives HIF-1/2a, which transcriptionally regulate numerous genes that play important roles in angiogenesis, metabolism, erythropoiesis, cell fate, survival, invasion and metastasis 17. It is reported that inducing the cell proliferation of breast cancer 18 and osteosarcoma cells 19 under CoCl_2_-simulated hypoxia is HIF-1 α dependent. However, it has been also demonstrated that synovial mesenchymal stem cells (SMSCs) 20 merely grow better under hypoxia along with upregulation of HIF-1 α. Moreover, other cells of (synovial, human renal carcinoma cell line as well as human cervical carcinoma cells) show depressed growth 21, 22. Therefore, the proliferation effect of HIF-1 α during exposure to hypoxia is cell type- and organ-specific.

Recently, HIF-2α has also been shown to be capable of regulating the transcription of genes that are regulated in hypoxia 23. HIF-2α has a more limited tissue distribution of expression, such as endothelium, kidney, heart, lungs, and small intestine 24. It has also been suggested to play an important role in cancer responses to hypoxia through the promotion of cell proliferation and migration 23. Interestingly, HIF-2 α promoted hypoxic cell proliferation of von Hippel-Lindau (VHL)-deficient carcinoma cell in a c-Myc-dependent or mTORC1 activation fashion, while HIF-1 α inhibited their growth 24, 25. In addition, other research showed that *in vivo* exposure to hypoxia leaded to a remarkable proliferation of bronchial epithelium 26 and carotid body 27. Thus, it will be interesting to further look into the interplay between HIF-2 α and hypoxia-induced cell proliferation of cholesteatoma keratinocytes.

To explore the potential mechanisms responsible for this enhanced proliferation, we focused on the PI3K-Akt signaling cascade. PI3K might be activated by growth factors, as well as hormones, and in turn mobilizes PDK1 along with PDK2 to the cell membrane, and then PDK1/PDK2 cooperate to activate Akt completely 3. Akt enhances cell survival via repressing apoptosis, and is also linked to the modulation of the cell cycle. Previous investigations have documented that the PI3K-Akt signaling cascade serves a crucial role in the hyperproliferation of cholesteatoma epithelial 3. Herein, we speculated the PI3K-Akt signaling cascade participates in the hypoxia‑mediated proliferation of cholesteatoma keratinocytes, and could be associated with hypoxia‑induced initiation and formation of cholesteatoma.

The *in vitro* study illustrated that the PI3K-Akt signaling cascade in cholesteatoma keratinocytes was remarkably activated by hypoxia. Herein, cholesteatoma keratinocytes revealed distinct potential to proliferate in response to hypoxia without exogenous growth factors. It was subsequently considered that the PI3K-Akt signaling cascade participates in hypoxia‑triggered proliferation behaviors of cholesteatoma keratinocytes. Specific inhibitor of the PI3K-Akt signaling cascade, LY294002 was employed to assess this, and it was illustrated that the hypoxia‑triggered proliferation was notably inhibited under the inhibitor treatment. This enhanced the evidence that the PI3K-Akt signaling cascade was critical for the hypoxia‑induced proliferation in cholesteatoma keratinocytes.

## Conclusions

The present study illustrated that hypoxia activates HIF‑1α and enhances cell proliferation through the PI3K-Akt pathways in cholesteatoma keratinocytes. These findings suggested that the tissue hypoxia encountered in pathophysiological conditions could contribute to the hypoxia-linked progression of the retraction pocket by promoting concurrent cellular growth. In addition, these data may be helpful for supporting the view that hypoxia plays a vital role in the formation of acquired cholesteatoma.

## References

[1] Zhang C, Liu YW, Chi Z, Chen B (2020). Ligand-Activated Peroxisome Proliferator-Activated Receptor beta/delta Facilitates Cell Proliferation in Human Cholesteatoma Keratinocytes. PPAR Res.

[2] Shih CP, Lee JT, Chen HK, Lin YC, Chen HC, Lin YY (2018). Comparison of changes in mitochondrial bioenergetics between keratinocytes in human external auditory canal skin and cholesteatomas from normoxia to hypoxia. Sci Rep.

[3] Xie S, Xiang Y, Wang X, Ren H, Yin T, Ren J (2016). Acquired cholesteatoma epithelial hyperproliferation: Roles of cell proliferation signal pathways. Laryngoscope.

[4] Sudhoff H, Tos M (2000). Pathogenesis of attic cholesteatoma: clinical and immunohistochemical support for combination of retraction theory and proliferation theory. Am J Otol.

[5] Tos M (1974). Production of mucus in the middle ear and Eustachian tube. Embryology, anatomy, and pathology of the mucous glands and goblet cells in the Eustachian tube and middle ear. Ann Otol Rhinol Laryngol.

[6] Adunka O, Gstoettner W, Knecht R, Kierner AC (2003). Expression of hypoxia inducible factor 1 alpha and Von Hippel Lindau protein in human middle ear cholesteatoma. Laryngoscope.

[7] Liu Y, Cui Y, Yu L, Zhang P (2005). [Expression of hypoxia-inducible factor-1alpha in middle ear cholesteatoma]. Lin Chuang Er Bi Yan Hou Ke Za Zhi.

[8] Darby IA, Hewitson TD (2016). Hypoxia in tissue repair and fibrosis. Cell Tissue Res.

[9] Xie Y, Shi X, Sheng K, Han G, Li W, Zhao Q (2019). PI3K/Akt signaling transduction pathway, erythropoiesis and glycolysis in hypoxia (Review). Mol Med Rep.

[10] Cantley LC (2002). The phosphoinositide 3-kinase pathway. Science.

[11] Osaki M, Oshimura M, Ito H (2004). PI3K-Akt pathway: its functions and alterations in human cancer. Apoptosis.

[12] Chai X, Sun D, Han Q, Yi L, Wu Y, Liu X (2018). Hypoxia induces pulmonary arterial fibroblast proliferation, migration, differentiation and vascular remodeling via the PI3K/Akt/p70S6K signaling pathway. Int J Mol Med.

[13] Alvarez-Tejado M, Alfranca A, Aragones J, Vara A, Landazuri MO, del Peso L (2002). Lack of evidence for the involvement of the phosphoinositide 3-kinase/Akt pathway in the activation of hypoxia-inducible factors by low oxygen tension. J Biol Chem.

[14] Chi Z, Wang Z, Liang Q, Zhu Y, du Q (2015). Induction of cytokine production in cholesteatoma keratinocytes by extracellular high-mobility group box chromosomal protein 1 combined with DNA released by apoptotic cholesteatoma keratinocytes. Mol Cell Biochem.

[15] Liu Y, Wang C, Wang Y, Ma Z, Xiao J, McClain C (2012). Cobalt chloride decreases fibroblast growth factor-21 expression dependent on oxidative stress but not hypoxia-inducible factor in Caco-2 cells. Toxicol Appl Pharmacol.

[16] Munoz-Sanchez J, Chanez-Cardenas ME (2019). The use of cobalt chloride as a chemical hypoxia model. J Appl Toxicol.

[17] Lee P, Chandel NS, Simon MC (2020). Cellular adaptation to hypoxia through hypoxia inducible factors and beyond. Nat Rev Mol Cell Biol.

[18] Rana NK, Singh P, Koch B (2019). CoCl2 simulated hypoxia induce cell proliferation and alter the expression pattern of hypoxia associated genes involved in angiogenesis and apoptosis. Biol Res.

[19] Zhang D, Cui G, Sun C, Lei L, Lei L, Williamson RA (2019). Hypoxia promotes osteosarcoma cell proliferation and migration through enhancing platelet-derived growth factor-BB/platelet-derived growth factor receptor-beta axis. Biochem Biophys Res Commun.

[20] Zheng W, Gu X, Sun X, Hu D (2019). Effects of hypoxiainducible factor1alpha on the proliferation and apoptosis of human synovial mesenchymal stem cells. Mol Med Rep.

[21] Cogo A, Napolitano G, Michoud MC, Barbon DR, Ward M, Martin JG (2003). Effects of hypoxia on rat airway smooth muscle cell proliferation. J Appl Physiol (1985).

[22] Shen C, Beroukhim R, Schumacher SE, Zhou J, Chang M, Signoretti S (2011). Genetic and functional studies implicate HIF1alpha as a 14q kidney cancer suppressor gene. Cancer Discov.

[23] Loboda A, Jozkowicz A, Dulak J (2010). HIF-1 and HIF-2 transcription factors-similar but not identical. Mol Cells.

[24] Gordan JD, Bertout JA, Hu CJ, Diehl JA, Simon MC (2007). HIF-2alpha promotes hypoxic cell proliferation by enhancing c-myc transcriptional activity. Cancer Cell.

[25] Elorza A, Soro-Arnaiz I, Melendez-Rodriguez F, Rodriguez-Vaello V, Marsboom G, de Carcer G (2012). HIF2alpha acts as an mTORC1 activator through the amino acid carrier SLC7A5. Mol Cell.

[26] Torres-Capelli M, Marsboom G, Li QO, Tello D, Rodriguez FM, Alonso T (2016). Role Of Hif2alpha Oxygen Sensing Pathway In Bronchial Epithelial Club Cell Proliferation. Sci Rep.

[27] Hodson EJ, Nicholls LG, Turner PJ, Llyr R, Fielding JW, Douglas G (2016). Regulation of ventilatory sensitivity and carotid body proliferation in hypoxia by the PHD2/HIF-2 pathway. J Physiol.

